# Preschool Externalizing Behavior Predicts Gender-Specific Variation in Adolescent Neural Structure

**DOI:** 10.1371/journal.pone.0117453

**Published:** 2015-02-06

**Authors:** Jessica Z. K. Caldwell, Jeffrey M. Armstrong, Jamie L. Hanson, Matthew J. Sutterer, Diane E. Stodola, Michael Koenigs, Ned H. Kalin, Marilyn J. Essex, Richard J. Davidson

**Affiliations:** 1 Department of Psychology, University of Wisconsin–Madison, Madison, Wisconsin, United States of America; 2 Department of Psychiatry, University of Wisconsin–Madison, Madison, Wisconsin, United States of America; 3 Center for Investigating Healthy Minds, University of Wisconsin–Madison, Madison, Wisconsin, United States of America; Technion - Israel Institute of Technology, ISRAEL

## Abstract

Dysfunction in the prefrontal cortex, amygdala, and hippocampus is believed to underlie the development of much psychopathology. However, to date only limited longitudinal data relate early behavior with neural structure later in life. Our objective was to examine the relationship of early life externalizing behavior with adolescent brain structure. We report here the first longitudinal study linking externalizing behavior during preschool to brain structure during adolescence. We examined the relationship of preschool externalizing behavior with amygdala, hippocampus, and prefrontal cortex volumes at age 15 years in a community sample of 76 adolescents followed longitudinally since their mothers’ pregnancy. A significant gender by externalizing behavior interaction revealed that males—but not females—with greater early childhood externalizing behavior had smaller amygdala volumes at adolescence (*t* = 2.33, *p* = .023). No significant results were found for the hippocampus or the prefrontal cortex. Greater early externalizing behavior also related to smaller volume of a cluster including the angular gyrus and tempoparietal junction across genders. Results were not attributable to the impact of preschool anxiety, preschool maternal stress, school-age internalizing or externalizing behaviors, or adolescent substance use. These findings demonstrate a novel, gender-specific relationship between early-childhood externalizing behavior and adolescent amygdala volume, as well as a cross-gender result for the angular gyrus and tempoparietal junction.

## Introduction

The amygdala, hippocampus, and prefrontal cortex (PFC), especially ventromedial and orbitofrontal regions, are key neural substrates for processing and regulating emotion and social behavior. Altered structure and function within this neural circuit have been associated with diverse psychopathologies, including externalizing [1–4] and internalizing [5,6] disorders. Recent neurodevelopmental research has highlighted adolescence as a critical period for maturation of prefrontal structure and limbic/prefrontal connectivity [7,8]. Further, rates of psychiatric disorders increase in adolescence [9,10]. Thus, a major goal for affective and cognitive neuroscience is to link prefrontal and limbic structure and function with symptoms of psychopathology across development.

Longitudinal studies offer unique opportunities to examine markers and underlying mechanisms of vulnerability to psychopathology. However, few neuroimaging studies have utilized cohorts followed from early childhood, and most of these have emphasized internalizing behaviors [11–16]. The single such study of childhood externalizing behavior showed a relationship between aggression across ages 7.5–11 and smaller adult male amygdala volume [16]. These findings complement cross-sectional studies of clinical antisocial behavior, which have most consistently implicated the amygdala and PFC [2,3,17,18], structures that are central in research on externalizing disorders. In particular, amygdala volume abnormalities have been suggested to underlie lower sensitivity to punishment and threat cues and reduced learning of socially reinforcing stimuli, and prefrontal differences to abnormalities in emotion regulation and prediction of consequences [19]. However, investigations have also implicated other structures in externalizing behavior, including the hippocampus [4,20,21], variations in which may contribute to reduced emotion-related learning [22]. Unexamined thus far is whether very early externalizing behaviors predict structural brain differences in adolescence, and whether these might be more significant predictors of later neural structure due to early brain plasticity and the documented role of early experience in shaping the brain [23]. It is also unknown whether these early behaviors explain later neural structure beyond contributions of early life internalizing behaviors, early life stress [24–26], later mental health symptoms, and substance use [27–30]. Moreover, despite well-established gender differences in externalizing behaviors [31,32], longitudinal neural correlates of female externalizing behaviors remain unexplored.

The present study examined the relationship of early-life externalizing behavior with adolescent amygdala, hippocampus, and PFC volumes in a community sample of 76 adolescents followed longitudinally since birth. We employed rigorous manual segmentation of the amygdala and hippocampus and whole-brain tensor-based morphometry, an analytic strategy that has yielded significant information on brain development [33,34]. Our primary hypothesis was that preschool externalizing behavior would be associated with smaller amygdala and PFC volumes at adolescence. The hippocampus was examined in an exploratory fashion, given previous mixed findings [4,21]. Next, we examined whether school-age externalizing behavior added to the explanatory power of early-life externalizing, with the expectation that early externalizing behavior would be more strongly associated with adolescent amygdala, hippocampus, and PFC volumes than later externalizing behavior. Finally, we considered other relevant variables including gender, maternal stress, preschool anxiety symptoms, school-age internalizing symptoms (i.e., anxiety and depression), school-age ADHD symptoms, and adolescent substance use. Overall, our aim was to add to the literature on neural correlates of early life externalizing behavior in males and females, and we achieved this aim.

## Materials and Methods

### Participants

A sample of 83 adolescents (45 female; mean age = 14.7 years) was recruited from the longitudinal Wisconsin Study of Families and Work [35]. While other aspects of this longitudinal study have been published, data contained here have not been published elsewhere. Original inclusion criteria were that mothers be over age 18, in the second trimester of pregnancy, living with the baby’s father, and employed or a full-time homemaker. Age 15 inclusion criteria were family’s ability to travel to the laboratory and standard MRI eligibility. The University of Wisconsin–Madison Institutional Review Board approved this research; participants’ parents gave written informed consent, and participants gave written informed assent. Research was conducted in accordance with principles expressed in the Declaration of Helsinki.

Post-scan exclusions yielded 76 subjects (42 female, mean age 14.7 years). Subjects were excluded for neural anomaly (1); history of infant febrile seizures (1); missing data, i.e., pubertal status (1), preschool externalizing (2), or more than 2 of the school-age assessments (1). One final data point was excluded due to a highly discrepant manually-traced amygdala volume thought to possibly relate to poor scan quality i.e., preliminary analyses, prior to the current manuscript, indicated this data point had studentized residual values > 4, which indicated a very significant outlier, and a Cook’s D value of nearly 0.5 (cutoff ∼0.05), indicating this point would significantly influence any further analyses (1).

### Preschool Externalizing Symptoms

Preschool externalizing symptoms were assessed with the Preschool Behavior Questionnaire (PBQ) [37] using an 11-item scale that covers a broad range of externalizing behaviors (e.g., Is disobedient; Tells lies; Fights with other children). Mothers, fathers, and another familiar adult rated child externalizing behaviors at age 4.5 years on a 3-point scale (Does not apply, Applies sometimes, or Frequently applies); scores were averaged across reporters.

### School-Age Externalizing Symptoms

School-age externalizing symptoms were assessed with multi-informant scores at child ages 7, 9, 11, 13, and 15 years. Adult- and child-report measures were developed in tandem to parallel one another. Mothers and teachers completed the MacArthur Health and Behavior Questionnaire (HBQ) [38,39]. Child report was obtained at age 7 via the Berkeley Puppet Interview [40] and subsequently with self-report HBQ [41]. Analyses employed the externalizing symptom scale from each measure, which covers oppositionality/defiance (6–9 items; e.g., Defiant, talks back to adults), conduct problems (9–15 items; e.g., Lies or cheats; Vandalizes), overt aggression (4–8 items; e.g., Gets in many fights), and relational aggression (6–7 items; e.g., Tries to get others to dislike a peer). At each age, principal components analysis (PCA) was used to create multi-informant scores [42]; to obtain an overall level of school-age symptoms as an assessment of trait-like externalizing behavior, PCA-derived scores were averaged across the 5 assessments and z-scored.

### Control Variables

**Preschool maternal stress.** Maternal stress scores consisted of a five-domain, PCA-derived, maternal-report composite at child age 4.5, which encompassed maternal depressive symptoms, parenting stress, and role overload; family expressed anger; and financial stress [43].

**Preschool anxiety symptoms.** Preschool anxiety was assessed with a 9-item PBQ scale (e.g., Is worried, worries about many things). As with PBQ externalizing, responses were averaged across mother, father, and other-familiar-adult ratings at child age 4.5.

**School-age mental health symptoms.** HBQ generalized anxiety (7–13 items; e.g., Nervous, high strung, or tense), depression (6–16 items; e.g., Unhappy, sad, or depressed), and inattention/impulsivity (10–18 items; e.g., Cannot concentrate, cannot pay attention for long; Impulsive or acts without thinking) were multi-informant scores, averaged over 5 time-points as for HBQ externalizing behavior.

**Adolescent pubertal status.** At child age 15, a comprehensive puberty score was created using adolescent report of Tanner stages [44,45] and mother report of the Petersen Pubertal Development Scale [46] (5-point scale; scores range from 1 = no development to 5 = development seems complete) [47,48].

**Adolescent substance use.** At age 15, participant-reported substance use over the past 30 days was assessed. For alcohol and tobacco separately, total use was estimated, i.e., number of days used multiplied by typical number of drinks or cigarettes per day [49]; summary variables were z-scored.

### MRI Parameters

High-resolution whole-brain anatomical images were collected using a GE 750 3T scanner (GE Medical Systems, Waukesha, WI, USA) (T1-weighted inversion recovery fast gradient echo, 124 axial slices, flip angle = 30°, Matrix = 256 x 192, FOV = 240, .9375x.9375x1.2 mm) and reconstructed using in-house software. Image inhomogeneities were smoothed with a multispectral segmentation/bias correction algorithm (FAST) [50], and images underwent skull and vessel correction, and contrast-adjustment. As in previous publications, images were reoriented to the “pathological plane” for more accurate comparison to atlases [51,52]. Specifically, images were first aligned to anterior commissure-posterior commissure (i.e., AC-PC) space and then rotated about the transverse axis such that in the mid-sagittal plane, the posterior junction of the tentorium of the cerebellum was aligned to the same horizontal plane as the inferior margin of the frontal lobe (i.e., as if the cerebrum were sitting on a microtome for sectioning).

### Amygdala and Hippocampus Region of Interest (ROI) Definition

Given high variability and low validity of automated segmentation methods for regions like the amygdala [53,54], we employed rigorous manual segmentation of the amygdala and hippocampus. ROIs were manually traced by highly trained staff, who were blind to participant information, using in-house software.

**Amygdala Region of Interest (ROI) Definition.** Definition was completed according to previously established protocol [51]. Briefly, the optic tract, optic radiations, hippocampus, and inferior horn of the lateral ventricle were used to define the posterior border; temporal lobe white matter, cerebrospinal fluid (CSF), the anterior commissure, and entorhinal cortex were used to define the anterior boundaries. Following initial tracing in the axial plane, traces were refined in coronal and sagittal planes by comparison with ex-vivo atlas sections [55,56]. In particular, the sagittal view was used to confirm accurate separation of the amygdala from hippocampus, entorhinal cortex, optic radiations, and caudate/putamen, while the coronal view was used for refinement of the dorsolateral and inferomedial boundaries [51].

Interrater and spatial reliability were assessed according to established protocol [51]. Specifically, volumetric reliability was assessed via tracing of 6 randomly selected images (12 amygdalae) and examination of interrater intraclass correlation (ICC), which equaled 0.91. Spatial reliability assessment was conducted to ensure that high interrater reliability was not an artifact of numerically similar but spatially divergent amygdala masks; this statistic (intersection/union) averaged 0.84 over the 6 images.

**Fig 1:**
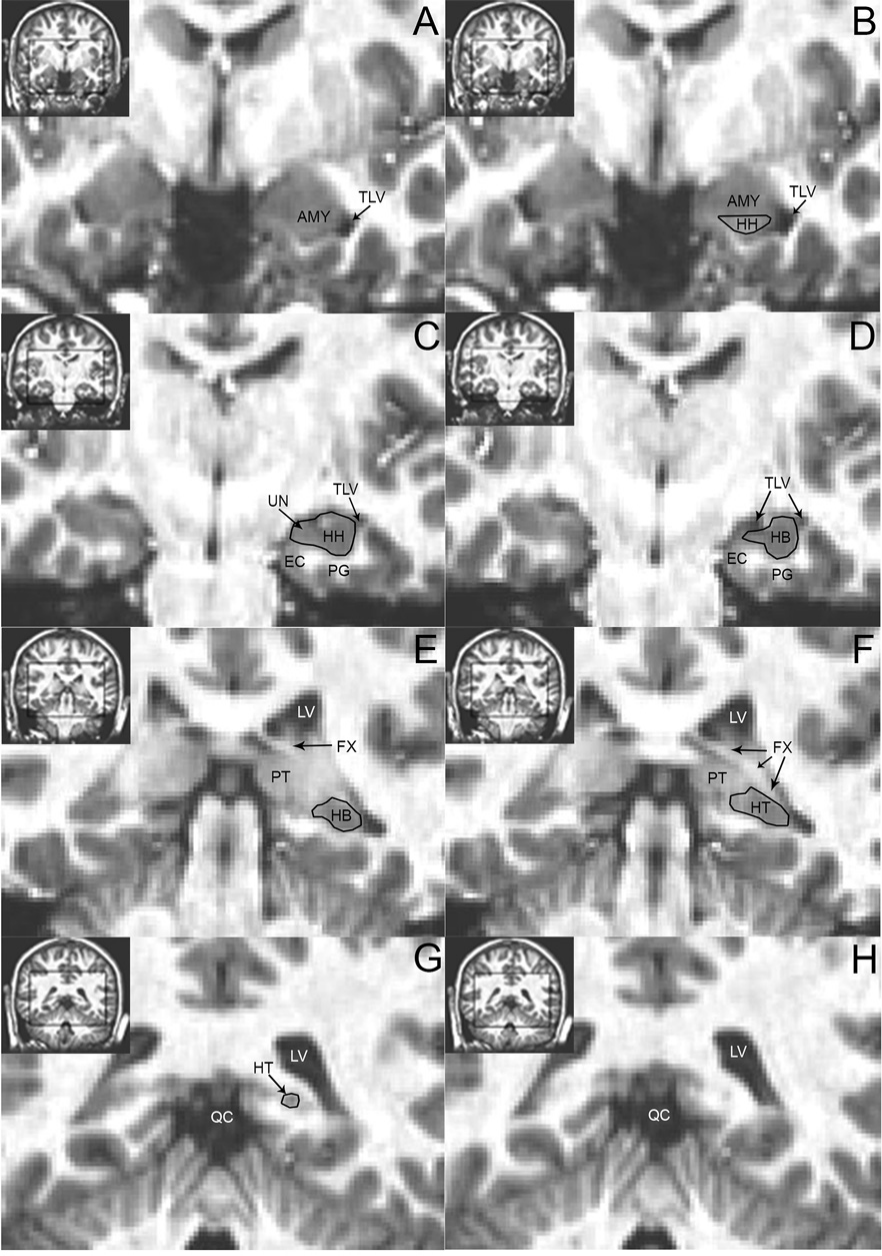
Hippocampus tracing landmarks as viewed on coronal native space images. Panels A-H, viewed from top to bottom and left to right in each row, show anterior to posterior progression through the hippocampus. Insets show areas of focus on full-brain sections (black box). An example region of interest is traced in black and labeled by hippocampal subregion: hippocampal head (HH), body (HB), and tail (HT). AMG = amygdala; FX = fornix; EC = entorhinal cortex; LV = lateral ventricle; PG = parahippocampal gyrus; PT = pulvinar of the thalamus; QC = quadrigeminal cistern; TLV = temporal horn of the lateral ventricle; UN = uncus.

**Hippocampus Region of Interest (ROI) Definition.** The most anterior aspect of the hippocampal head (HH) was defined as the coronal slice where the temporal horn of the lateral ventricle (TLV) appeared and the alveus was present (Fig. 1). Sagittal view was used for more accurate identification of the alveus (Fig. 1 A-B). The superior border to the HH was identified either by the ventricle arching above it or the white matter of the alveus, separating HH from amygdala. Where decreased image resolution or contrast prevented using these landmarks, the HH was defined superiorly by an arbitrary horizontal line from the superior margin of the TLV to the ambient cistern, in coronal view. The inferior border of the HH was defined as the white matter separating HH from parahippocampal gyrus. Laterally, the HH was defined by the TLV. In more anterior aspects of the HH, the medial border of the HH was defined by an arbitrary vertical line extending from the most medial aspect of the parahippocampal gyrus white matter to the ambient cistern. This line was used to separate the HH from the uncus, which consists mostly of entorhinal cortex and amygdala in this area. Beginning in the most anterior coronal slice where the uncal sulcus separates the hippocampus portion of the uncus from entorhinal cortex, the uncus was included in the HH; here the ambient cistern defined the HH medially.

The hippocampal body (HB) was defined anteriorly as the coronal slice in which the uncus was no longer present (Fig. 1 C-D). Where the uncus was difficult to distinguish from the hippocampus, three hierarchical criteria were used to identify the first slice of the HB: clearance of tissue from the ambient cistern and most medial section of the TLV, a more circular shape and lateral position of the hippocampus, and thinning of the inferomedial gray matter. Sagittal and axial views confirmed the HH/HT border. For all aspects, the HB followed the same general criteria as the HH, i.e., lateral, superior and medial borders were defined by the CSF of the TLV and ambient cistern; inferior border was defined by white matter. Careful attention was given to the superior HB border to avoid inclusion of the tail of the caudate, lateral geniculate and stria terminalis.

The anterior aspect of the hippocampal tail (HT) was defined generally following Malykhin’s guidelines [57]. In an effort to include as much tissue as possible in the HT, its anterior border was defined as the slice displaying at least 75% of the full profile of the crus of the fornix (Fig. 1 E-F). Similar to the HH and HB, the HT was defined as bordered laterally by the lateral ventricle, medially by either CSF or white matter, inferiorly by white matter, and superiorly by either lateral ventricle or white matter. Care was taken to avoid including the pulvinar of the thalamus and tail of caudate on the superior border. White matter of the fornix was excluded. Fasciolar gyrus and Andreas Retzius gyrus were included [58]. The posterior aspect of the HT was identified as the coronal slice where grey matter starts to appear inferiomedially to the lateral ventricle [59] (Fig. 1 G-H).

As for the amygdala, interrater and spatial reliability were assessed following established procedures [51,60,61]. Specifically, raters traced 5 randomly selected images (10 hippocampi); ICC was 0.97, and spatial reliability averaged 0.81.

### Tensor-Based Morphometry (TBM)

Whole-brain TBM was employed to examine prefrontal associations, using Advanced Normalization Tools (ANTS) [62], one of the best available nonlinear warping algorithms [63], and FMRISTAT [64]. TBM is well-suited for a pediatric population, as it minimizes sources of variability such as brain tissue segmentation, uses a study-specific anatomical template, and yields high sensitivity at the voxel level.

**Imaging processing and template creation.** T1-weighted images were corrected for field inhomogeneity, masked to include brain tissue and ventricular CSF, and used to construct an optimal, study-specific template, via a diffeomorphic shape and intensity averaging technique [62,65]. The region-based cross-correlation similarity metric was employed. Processing yielded an unbiased average shape and appearance template and the set of diffeomorphisms and inverse diffeomorphisms that map from the template to each individual.

**Symmetric diffeomorphic image normalization and tensor-based morphometry.** After affine transformation, each individual brain was registered to the template using symmetric normalization available in the Advanced Normalization Tools (ANTS) package [62]. Symmetric normalization allows for large, yet physically reasonable, deformations and produces deformation tensor fields, defined in the template space, which chart voxelwise shape change from the template to each subject’s brain.

Jacobian determinants of the deformation field indicate fractional volume expansion and contraction at each voxel, quantifying magnitude of volume alterations required to match the template. Jacobian determinants were log transformed to increase proximity to the normal distribution [62] and smoothed using a Gaussian filter, yielding statistical maps with 8 mm at full-width, half-maximum spatial smoothness.

### Data Analysis

**Manually-segmented amygdala and hippocampus analyses.** Kolmogorov-Smirnov tests confirmed normality for all variables. Amygdala and hippocampus ROI analyses employ residual volumes after regressing out cerebrum volume (i.e., total brain volume excluding brainstem and cerebellum) and pubertal status. Prior to analyses central to our hypotheses, we examined validity of evaluating the amygdala and hippocampus separately by brain hemisphere, and the hippocampus by subregion. A repeated measures general linear model (GLM) with hemisphere as a within-subjects factor and pubertal status and total brain volume as covariates showed no effect of hemisphere for either structure; therefore, findings are presented for the mean of left and right ROI volumes. Similarly, a repeated measures GLM with hippocampus subregion (i.e., head, body, and tail) as a within-subjects factor and gender and preschool externalizing as between-subjects factors showed no significant interaction of gender, externalizing, and subregion. Thus, total hippocampus volumes were employed.

Subsequently, linear regression analyses examined the relationships of preschool externalizing behavior with adolescent ROI volumes using centered variables. For each regression, gender and preschool externalizing, as well as the interaction of gender and preschool externalizing behavior, were entered as predictors of adolescent ROI volumes. A second hierarchical regression examined the additional contribution of school-age externalizing behavior by entering gender, preschool externalizing behavior, and the interaction of gender and preschool externalizing behavior in the first step and school-age externalizing behavior in a second step. Significant findings for preschool externalizing behavior were followed up with a hierarchical regression with gender, preschool externalizing, and the interaction of gender and preschool externalizing behavior predicting ROI volume in the first step, and potential confound variables (i.e., maternal stress at child age 4.5; preschool anxiety; school-age depression, anxiety, and ADHD symptoms; and adolescent alcohol use and tobacco use) entered via stepwise inclusion in a second hierarchical step. As these latter analyses revealed no significant contribution from any potential confounders, confound variables are not considered further.

Significant regression analyses were also subjected to regression diagnostics to assess for outliers of significant influence. First, the regression was run as above, saving studentized residual values and Cook’s Distance. Outliers were defined as data points with studentized residuals > 2. Outliers of influence were defined as data points with studentized residuals > 2 and Cook’s Distance > (4/N-k-1) cutoff; N = sample size; k = number of predictors) [36].

**Tensor-based morphometry.** Regression models were constructed examining behavioral variables of interest and variations in brain structure across the whole brain in FMRISTAT [64] for the full sample and for males and females separately. Whole-brain volume and pubertal status were entered into a linear regression model as nuisance variables. As in other work [25], an initial statistical threshold of p < .005 uncorrected was used in examining possible brain differences in relation to preschool externalizing behaviors. Signal above this threshold was corrected using Gaussian random-field theory [66] to limit type I error.

## Results

### Descriptive Statistics

**Fig 2:**
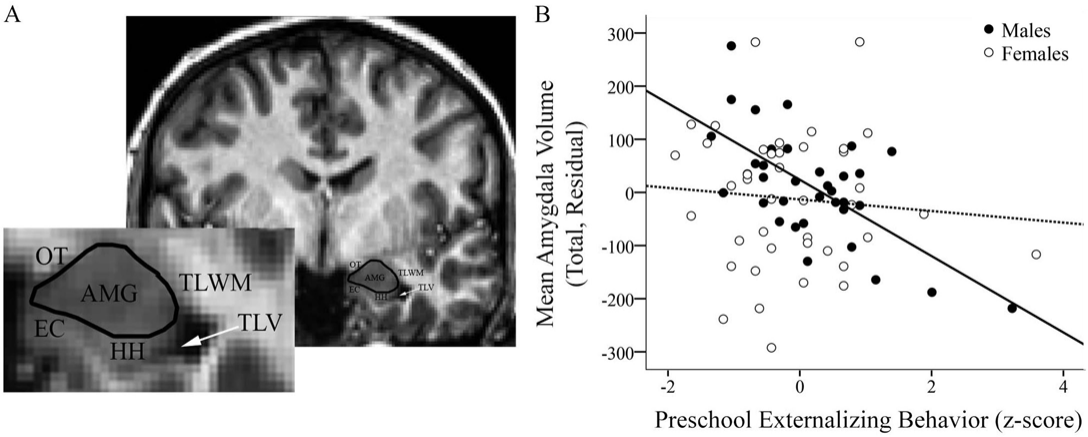
Greater externalizing behavior in preschool correlates with smaller male amygdala volume. A) Representative coronal view of the amygdala with key neuroanatomical landmarks denoted: AMG = amygdala; EC = entorhinal cortex; HH = hippocampal head; OT = optic tract; TLV = temporal horn of the lateral ventricle; TLWM = temporal lobe white matter. B) Correlation of preschool externalizing with amygdala volumes (upper panel: male r = -0.653, *p* = 0.000029; female r = -0.088, *p* = 0.578).

Participant pubertal status at age 15 was rated as nearly complete (mean = 4.47 on a 1–5 scale; SD = 0.48), with female development more advanced (Mann-Whitney *z* = -3.425; *p* = .001). Preschool externalizing scores had a mean of 5.18 (SD = 2.74) out of 22 possible points; gender differences were not significant (Mann-Whitney *z* = -1.642; *p* = .101). School-age externalizing z-scores had a mean of -0.0014 (SD = 0.872); gender differences were not significant (Mann-Whitney *z* = -1.740; *p* = .082). The level and variance of externalizing scores observed are consistent with other community samples in preschool [37,67] and elementary school [68,69], with scores covering a range that includes low, subclinical, and clinical levels. Correlations of ROI volumes with externalizing variables are presented in Table 1 and Fig. 2B. Descriptive statistics for raw whole brain volumes and amygdala and hippocampus ROI volumes are presented in Table 2.

**Table 1:** Pearson correlations of neural volumes and externalizing behaviors.

Males	1	2	3
1. Mean Amygdala Volume			
2. Mean Hippocampus Volume	0.327		
3. Preschool Externalizing	-0.653^a^	-0.105	
4. School-Age Externalizing	-0.276	-0.018	0.291
			
Females	1	2	3
1. Mean Amygdala Volume			
2. Mean Hippocampus Volume	0.268		
3. Preschool Externalizing	-0.088	0.294	
4. School-Age Externalizing	-0.173	0.008	0.684^a^
			

Amygdala and hippocampus volumes have been corrected for total brain volume and pubertal status. Externalizing behaviors have been standardized (z-scores).

^a^ *p* < 0.01

**Table 2:** Descriptive statistics for raw computed and hand-traced neural volumes.

	Volume Mean and SD (mm^3^)		
Region	Males (N = 34)	Females (N = 42)	Full Sample (N = 76)
Whole Brain	1.20 E 6 (1.01 E-5)	1.05 E 6 (7.20 E-4)	1.12 E 6 (1.15 E-5)
Left Amygdala	1820.47 (122.51)	1718.69 (151.30)	1764.22 (147.35)
Right Amygdala	1832.18 (128.36)	1729.06 (132.79)	1775.19 (139.83)
Left Hippocampus	2861.00 (328.45)	2474.14 (342.48)	2647.21 (386.11)
Left Hippocampus Head	1487.94 (267.15)	1237.31 (246.75)	1349.43 (283.59)
Left Hippocampus Body	964.32 (174.86)	878.90 (149.18)	917.12 (165.67)
Left Hippocampus Tail	409.03 (102.95)	358.14 (107.62)	380.91 (107.90)
Right Hippocampus	2928.29 (294.31)	2530.79 (313.89)	2708.62 (362.71)
Right Hippocampus Head	1566.94 (228.18)	1325.60 (237.97)	1433.57 (261.64)
Right Hippocampus Body	939.88 (163.87)	840.26 (150.17)	884.83 (163.19)
Right Hippocampus Tail	421.59 (122.69)	365.21 (88.45)	390.43 (108.15)

Whole brain volumes (mm^3^) were segmented automatically; all other volumes are uncorrected hand-traced volumes (mm^3^). See Statistical Analysis section in Materials and Methods for description of how raw amygdala and hippocampus volumes were corrected for total brain volume and pubertal status.

### Preschool Externalizing Behavior and Amygdala Volume

We examined the hypothesis that preschool externalizing would be associated with smaller adolescent amygdala volume. A linear regression showed main effects for preschool externalizing behavior, such that higher preschool externalizing predicted smaller amygdala volumes (t = -3.161, *p* = .002). The effect of gender was not significant (t = -1.444, *p* = .153). Results revealed a significant gender by externalizing behavior interaction (t = 2.328, *p* = .023). Males, but not females, with greater preschool externalizing had smaller adolescent amygdala volumes (see Fig. 2A, B). Although regression diagnostics identified two outlying cases based on studentized residual values >2, and one case that also met influence criteria due to Cook’s D > (4/N-k-1) cutoff, the exclusion of that case did not reduce any findings to non-significance, thus this case was retained.

To determine whether school-age externalizing behaviors contribute to prediction of neural structure beyond what is explained by early-life externalizing behaviors, a second hierarchical regression analysis was completed with preschool externalizing predicting amygdala or hippocampus volume in the first step and school-age externalizing behaviors entered using stepwise inclusion in a second step. School-age externalizing did not contribute significant additional variance. As noted in Materials and Methods, no control variable—including preschool anxiety—explained significant variance.

### Preschool Externalizing Behavior and Hippocampus Volume

Next, we examined the relationship of preschool externalizing with adolescent hippocampus volumes. Results revealed a main effect of gender, such that females had significantly smaller hippocampal volumes compared to males (t = -2.351, *p* = .021), but no main effect of externalizing (t = 0.796, *p* = 0.428). The interaction of gender and externalizing was not significant (t = 1.699, *p* = .094). Regression diagnostics showed three outlying cases based on studentized residual value >2; however, none of these three cases was of significant influence based on Cook’s D value, thus cases were retained.

### Preschool Externalizing Behavior and Prefrontal Cortex

**Fig 3:**
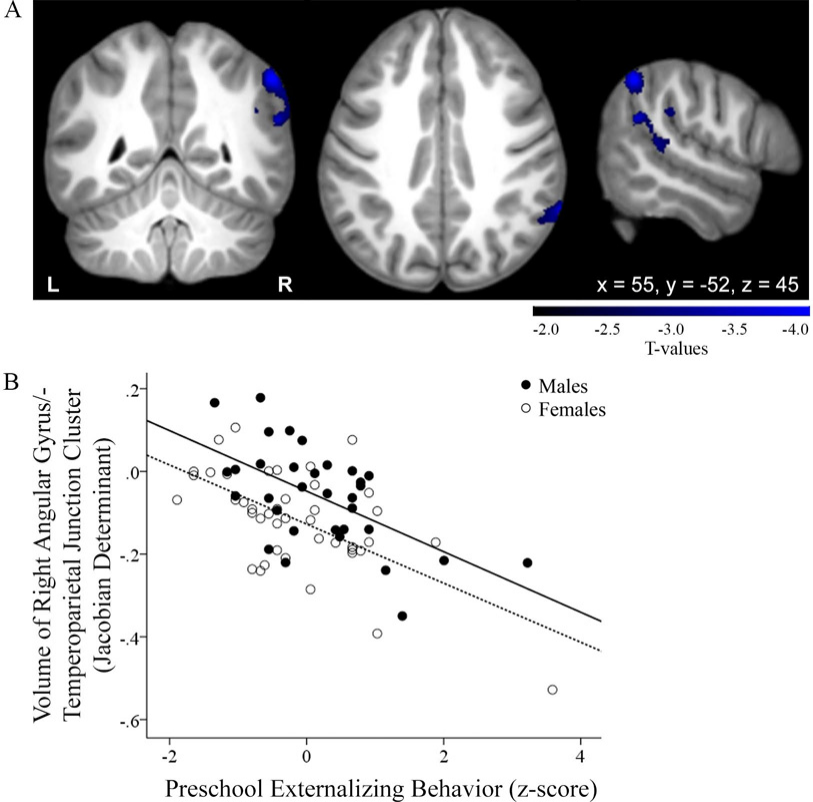
Greater preschool externalizing behavior relates to smaller right angular gyrus and temporoparietal junction, across genders. A) Coronal, sagittal, and axial views; coordinates indicate cluster peak, within the angular gyrus. B) Correlation of individual subject cluster volume and preschool externalizing behavior (full sample: r = -0.536, *p* = 0.000000705; male: r = -0.584, *p* = 0.000355; female r = -0.607, *p* = 0.0000199).

Voxelwise whole-brain regressions tested the hypothesis that early externalizing behavior would be associated with reduced PFC volume. No significant association with any PFC region was observed. The single significant association that emerged showed that greater preschool externalizing symptoms predicted smaller volumes within a cluster including right angular gyrus and temporoparietal junction (peak value: r = -4.18; *p* = 0.002; peak MNI coordinates: x = 55, y = -52, z = 45) (Fig. 3A, B). There were no significant results for the interaction of gender and externalizing behavior.

## Discussion

The current investigation extends literature associating early-life behavior with adolescent and adult brain structure, focusing uniquely on preschool externalizing behavior in a community sample. Analyses employing manual segmentation of adolescent amygdala and hippocampus showed an association of greater preschool externalizing behavior with smaller male amygdala volumes, but no significant results for the hippocampus. Tensor-based morphometry analyses showed no relationship of preschool externalizing behavior with adolescent PFC volume, but revealed greater early externalizing behavior related to smaller volume of a region including the angular gyrus and temporoparietal junction, across genders. Other potentially relevant variables, including preschool maternal stress, preschool anxiety, school-age internalizing and externalizing behavior, and adolescent substance use did not account for these findings.

The observed relationship of greater preschool externalizing with smaller adolescent male amygdala volume is consistent with the single previous longitudinal investigation of childhood externalizing behavior and adult brain volume in a clinical sample [16] and also with work in a sample at high familial risk for externalizing behavior [70]. This result further supports cross-sectional investigations with samples of conduct-disordered children and adolescents [71–73] and adults with a history of clinical externalizing [74,75]. Thus, the current study adds to previous work by showing measurable differences in a region important for emotion processing and social learning [76–78], even in a community sample with generally modest levels of early-life externalizing behaviors, as early as age 4.5 years.

This very early life externalizing behavior predicted adolescent amygdala volume beyond the impact of later behavior. While consistent with amygdala sensitivity to early experience [23], the implications of this finding will require further investigation. Longitudinal neuroimaging studies are needed to determine when in the course of development the variations in amygdala volume first emerge and whether such variations represent negative consequences of, adaptive responses to, or risk factors for externalizing behaviors [4,79]. Future work should also examine the role of functional amygdala responses and behavioral differences influenced by the amygdala, such as sensitivity to punishment and threat, in the relationship between amygdala structure and externalizing behavior [80]. It is possible that early life behavior is particularly relevant to later brain structure in a community sample with primarily non-clinical externalizing behavior; it is an open question whether this pattern might be seen in samples of clinical externalizing disorders, or whether in those samples later trait behavior is equally or more predictive.

Of note, the present amygdala findings also complement longitudinal research relating early inhibited temperament—which has been linked to internalizing symptoms—to larger adult amygdala volume [14], and in doing so highlight overlap in neural circuits associated with childhood internalizing and externalizing disorders. Given the complex structure of the amygdala [81] and its role in emotion and behavior, it may be fruitful to examine timing, directionality, and degree of structural amygdala variation and the role this plays in comorbidity [82,83]. Gender may be particularly important to consider in this pursuit, given gender differences in prevalence and course of internalizing and externalizing disorders [84,85] as well as in neural development, structure, and function [32,86–88].

Contrary to our a priori hypothesis, we did not find an association between externalizing behavior and smaller volume within the PFC using tensor-based morphometry. We did however uncover a relationship between greater early externalizing and smaller volumes near the right angular gyrus and temporoparietal junction, across genders. This finding is consistent with studies showing decreased temporal lobe [89–91] and parietal volumes [71] in clinical samples and complements research related to clinical externalizing, including studies implicating the angular gyrus in making moral judgments [92] and the temporoparietal junction in decoding others’ emotional states [93] and acting altruistically [94]. In contrast, the present TBM results were not consistent with research showing reduced PFC (i.e., ventromedial PFC, orbitofrontal cortex) in clinical samples [2,3,95]. It is also noteworthy that TBM analyses did not uncover amygdala volume differences detected with manual segmentation. We believe this discrepancy is due to the greater precision of manual segmentation and possibly due to testing of the manually segmented amygdala as an a priori region of interest, thus not requiring correction for multiple comparisons.

Although several studies have linked variability in hippocampal volume with externalizing behavior [4,20,21], the present study did not. This null finding may suggest specificity for the relationship of early externalizing behavior with amygdala, but not hippocampal, volume at adolescence. Alternatively, effects may have reached significance with a larger sample or one with higher levels of severe externalizing behavior.

Several limitations of the current investigation deserve note. Particularly, the early behaviors studied here are not equivalent to or predictors of violent or clinically aggressive behaviors or psychopathic personality, and care must be taken not to equate these two phenotypes. The present study also cannot speak to causality—it remains an open question whether early-life behaviors are a symptom of early neural abnormalities, or a risk factor for neural dysregulation later in development. In addition, we cannot rule out that differences between our work and previous studies relate to differences in methodological approach to segmentation.

In summary, the present study provides unique evidence for a relationship between early childhood externalizing behavior and adolescent amygdala volume in males, as well as angular gyrus and temporoparietal junction volume across genders. This work augments existing literature on externalizing behavior by examining the relationship between early life externalizing and adolescent brain structure in a community sample. Strengths include longitudinal design, rigorous manual segmentation of key subcortical structures, TBM with particular strengths for pediatric populations, multi-informant measures of externalizing, and inclusion of both genders. Much future investigation will be required to clarify and expand our knowledge of externalizing syndromes and their neural correlates across genders.
